# Eu(O_2_C‐C≡C‐CO_2_): An Eu^II^ Containing Anhydrous Coordination Polymer with High Stability and Negative Thermal Expansion

**DOI:** 10.1002/chem.201904966

**Published:** 2020-02-11

**Authors:** Verena K. Gramm, Daniel Smets, Ireneus Grzesiak, Theresa Block, Rainer Pöttgen, Markus Suta, Claudia Wickleder, Thomas Lorenz, Uwe Ruschewitz

**Affiliations:** ^1^ Department of Chemistry University of Cologne Greinstraße 6 50939 Köln Germany; ^2^ Institut für Anorganische und Analytische Chemie WWU Münster Corrensstraße 30 48149 Münster Germany; ^3^ Anorganische Chemie Universität Siegen Adolf-Reichwein-Straße 57068 Siegen Germany; ^4^ current affiliation: Debye Institute for Nanomaterials Science Universiteit Utrecht Princetonplein 1 3584 CC Utrecht The Netherlands; ^5^ Institute of Physics II University of Cologne Zülpicher Straße 77 50937 Köln Germany

**Keywords:** acetylenedicarboxylate, coordination polymers, europium, Mössbauer spectroscopy, negative thermal expansion

## Abstract

Anhydrous Eu^II^–acetylenedicarboxylate (EuADC; ADC^2−^ = ^−^O_2_C‐C≡C‐CO_2_^−^) was synthesized by reaction of EuBr_2_ with K_2_ADC or H_2_ADC in degassed water under oxygen‐free conditions. EuADC crystallizes in the SrADC type structure (*I*4_1_/*amd*, *Z=*4) forming a 3D coordination polymer with a diamond‐like arrangement of Eu^2+^ nodes (*msw* topology including the connecting ADC^2−^ linkers). Deep orange coloured EuADC is stable in air and starts decomposing upon heating in an argon atmosphere only at 440 °C. Measurements of the magnetic susceptibilities (*μ*_eff_=7.76 μ_B_) and ^151^Eu Mössbauer spectra (*δ*=−13.25 mm s^−1^ at 78 K) confirm the existence of Eu^2+^ cations. Diffuse reflectance spectra indicate a direct optical band gap of *E*_g_=2.64 eV (470 nm), which is in accordance with the orange colour of the material. Surprisingly, EuADC does not show any photoluminescence under irradiation with UV light of different wavelengths. Similar to SrADC, EuADC exhibits a negative thermal volume expansion below room temperature with a volume expansion coefficient *α*_V_=−9.4(12)×10^−6^ K^−1^.

## Graphical abstract

**Multifaceted**! Orange coloured Eu(C_4_O_4_), the anhydrous Eu^II^ salt of acetylenedicarboxylic acid, has many interesting properties: it is stable in air for months, and upon heating in an inert atmosphere decomposition only starts at 440 °C. Below room temperature it shows a negative thermal volume expansion (NTE) and although containing Eu^II^ it does not exhibit any luminescence even at 10 K.

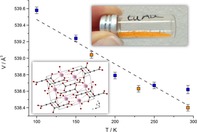

## Introduction

**Figure 1:**
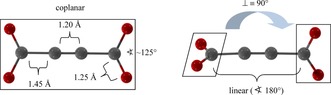
Two possible conformations of the acetylenedicarboxylate linker (ADC^2−^).

Among the many ligands that have been used for the construction of coordination polymers (CPs) and metal‐organic frameworks (MOFs) acetylenedicarboxylate (^−^O_2_C‐C≡C‐CO_2_^−^, ADC^2−^) belongs to the simplest ones. It consists of a short, rigid and linear C_4_ carbon backbone with a length of approx. 4.1 Å and two donating carboxylate groups (see Figure 1). Due to the free rotation around the C−C single bond, these carboxylate groups can adopt all torsion angles between a coplanar (0°, *D*_2*h*_ symmetry) and perpendicular arrangement (90°, *D*_2*d*_ symmetry). This is in contrast to the terephthalate linker (BDC^2−^), which is widely used for the synthesis of MOFs like MOF‐5^1^ or MIL‐53.^2^ For BDC^2−^ torsion angles between the carboxylate groups and the phenyl ring close to zero are found in most compounds.^3^ The length of the carbon backbone in the BDC^2−^ linker is approx. 5.75 Å. So, it is not surprising that many MOFs with permanent porosity were found with the latter, but only very few examples with the ADC^2−^ linker.^4^ Next to spatial restrictions also the low thermal stability of acetylenedicarboxylic acid (H_2_ADC) under solvothermal conditions, which are most widely used for the synthesis of porous MOFs,^5^ hinders the preparation of these materials. Very recently, Janiak and co‐workers used this low stability for an in situ hydrohalogenation of H_**2**_ADC and a straight‐forward synthesis of chlorofumarate based MOFs with MOF‐801 topology.4a, 4b

However, as pointed out by Cheetham and Rao, “apart from porous hybrid frameworks, the field has other opportunities to offer.”^6^, ^7^ Accordingly, numerous non‐porous CPs with ADC^2−^ ligands were synthesized with interesting magnetic,^8^ synthetic,^9^ luminescence^10^ or conductivity^11^ properties. In all examples additional ligands, mainly *N*‐donor ligands or water molecules, were used to crystallize these interesting materials. On the other hand, the field of anhydrous homoleptic CPs with the ADC^2−^ ligand is extremely small. At the moment only reports on the synthesis and crystal structure of SrADC,^12^ PbADC,^13^ CaADC,^14^ and Li_2_ADC^15^ are available. All compounds with composition M^II^ADC crystallize in the SrADC type structure (*I*4_1_/*amd*, no. 141, *Z=*4) with a diamond‐like arrangement, that is, the metal cations are arranged in an almost cubic diamond‐like fashion. The ADC^2−^ ligands connect these cations resulting in a M^II^O_8_ coordination sphere. It is remarkable that the carbon backbones of these linkers are all aligned along the tetragonal [001] axis. Thus, a tetragonal symmetry results, but the metric of the unit cell is still close to cubic ($\sqrt{2}$ *a*_tet_≈*c*_tet_).^12^ For SrADC, a negative thermal volume expansion (NTE) below room temperature was reported.^12^ The resulting thermal expansion coefficient is rather small (*α*_V_=−4.7(13) *x* 10^−6^ K^−1^) and by a factor of ≈6 smaller than the corresponding coefficients in other well‐known NTE materials like ZrW_2_O_8_ (*α*_V_=−27.2×10^−6^ K^−1^).^16^ The occurrence of NTE in SrADC was explained by a “guitar string vibration” of Sr⋅⋅⋅O⋅⋅⋅Sr units thus reducing the Sr⋅⋅⋅Sr distances with increasing temperature and increasing transverse vibrational motion of the bridging oxygen atom.^12^ Kepert and co‐workers developed a model for NTE in a number of Prussian Blue analogues, M^II^Pt^IV^(CN)_6_ with M^II^=Mn, Fe, Co, Ni, Cu, Zn, Cd.^17^ Here, the absolute values of *α*_V_ increase with increasing size of the M^II^ cation. This was correlated to the strengths of the metal‐cyanide bonding interaction leading to different energies of the transverse vibration of the cyanide bridge (i.e., “guitar string vibrations”).^17^ Accordingly, enhanced NTE behaviour is expected for more flexible structures.

To prove these concepts we aimed at the synthesis of EuADC, which is expected to crystallize isotypically to SrADC due to the very similar ionic radii of Eu^2+^ and Sr^2+^.^18^ However, because of the oxidation sensitivity of Eu^II^, the synthetic procedure for SrADC being performed in water and air could not simply be transferred to the respective Eu^II^ compound and a new approach had to be developed.

## Results and Discussion

### Synthesis of EuADC

Eu^II^ as a mild reducing agent (*ϵ*_0_(Eu^II^/Eu^III^)=−0.35 V) is oxidized in air to form Eu^III^. Therefore, in first experiments we reacted the electride of Eu in liquid ammonia with H_2_ADC under inert conditions to avoid contact with water and oxygen. The yellow‐orange precipitate that formed after addition of an equimolar amount of H_2_ADC to the blue electride was investigated by XRPD (Figure S1, Supporting Information). The comparison with a calculated pattern for SrADC showed that isotypic EuADC had formed. However, the reflections are very broad pointing to a low crystallinity of the material. Annealing at mild temperatures did not improve the crystallinity significantly and elemental analysis revealed up to 2 % nitrogen obviously due to NH_3_ that could not be removed completely.

Samples with a high crystallinity and sharp reflections were obtained by the reaction of K_2_ADC with EuBr_2_ in degassed water (room temperature, Argon atmosphere) according to Equation (1):

(1) $$\mathrm{EuBr}{}_{2}+K{}_{2}\mathrm{ADC}\to \mathrm{EuADC}+2\;\mathrm{KBr}$$

**Figure 2:**
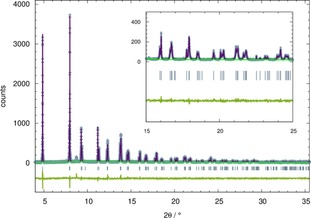
Rietveld refinement of the diffraction pattern of EuADC (SNBL, ESRF Grenoble/France, *λ*=0.504477 Å). Experimental data points (blue crosses), calculated profile (purple solid line) and difference curve (green curve below) are shown. Blue vertical bars mark the positions of Bragg reflections and the background is given as a turquoise solid line.

The orange precipitate was washed with degassed water, but even after several washing cycles small amounts of KBr remained in the final material (cp. weak reflection at 2*θ*≈8.7° in Figure 2). From a Rietveld refinement an amount of 3.5 weight % KBr was determined. A single phase sample was obtained by the reaction of H_2_ADC with EuBr_2_ in degassed water (room temperature, Ar atmosphere) according to Equation (2):

(2) $$\mathrm{EuBr}{}_{2}+H{}_{2}\mathrm{ADC}\to \mathrm{EuADC}+2\;\mathrm{HBr}$$

This also orange‐coloured product (Figure S2, Supporting Information) was used for all measurements reported in the following (with the exception of the synchrotron powder diffraction data recorded at the ESRF, where samples obtained according to Equation (1) were used). EuADC is stable in air. Even after several months no signs of degradation were detectable.

### Crystal Structure of EuADC

In contrast to SrADC^12^ no single crystals suitable for an X‐ray structure analysis were obtained for EuADC in the syntheses described above. Therefore, high‐resolution synchrotron powder diffraction data was used to refine the crystal structure of EuADC. It crystallizes isotypically with SrADC.^12^ The latter was used as a starting model for the refinement. In Figure 2 the plot of the Rietveld fit is shown confirming a very good agreement between the observed and refined data. Details of this refinement as well as some structural details are summarized in Table 1, fractional atomic coordinates and selected interatomic distances are given in Table S1 (Supporting Information).

**Table 1:** EuADC: Selected crystallographic data and some refinement details of the powder diffraction measurement at 295(2) K.

	EuC_4_O_4_
crystal system	tetragonal
space group, *Z*	*I*4_1_/*amd* (no. 141), 4
*M*_r_ [g mol^−1^]	264.00
*a* [Å]	7.21668(8)
*c* [Å]	10.3165(1)
*V* [Å^3^]	537.29(2)
*R*_p_	0.103
w*R*_p_	0.153
*R*_Bragg_	0.054
*χ* ^2^	1.04
data points	15 751
no. of refined parameters	25
no. of reflections	150
no. of restraints	3
background	shifted Chebyshev, 6 terms
data range [°]	4.0≤2*θ*≤35.5
step size [°]	0.002
instrument	SNBL/ ESRF
radiation, wavelength [Å]	synchrotron, *λ*=0.504477
CCDC deposition number^19^	1897411

**Figure 3:**
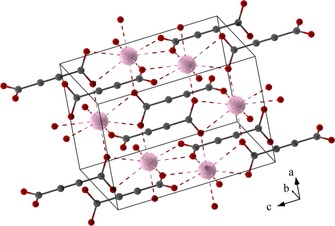
View of the crystal structure of EuADC. Short Eu^II^‐O distances are marked with dotted lines. Eu^II^ cations are drawn in pink, oxygen and carbon atoms in red and dark grey.

The resulting crystal structure of EuADC is depicted in Figure 3. It shall only be briefly discussed, as an extended description was already presented for isotypic SrADC.^12^ The unit cell volume of EuADC is slightly smaller than that of SrADC^12^ (cp. Table 3). The Eu^II^ cations form a diamond‐like arrangement with four identical Eu‐Eu distances (4.43531(4) Å) and Eu‐Eu‐Eu angles very close to the ideal tetrahedral angles: 109.76° (4×) and 108.89° (2×). These Eu^II^ cations are connected to each other by the ADC^2−^ linkers. It is remarkable that the carbon backbones of these linkers are all aligned parallel to [001] (Figure 3). This leads to a tetragonal symmetry, but the cubic symmetry is “preserved” by a *c*/*a* ratio very close to $\sqrt{2}\bar{}$ (cp. Table 3). Note that *I*4_1_/*amd*, the space group of the crystal structure of EuADC, is a direct subgroup (*translationengleich*, index 3) of *Fd* $\bar{3}$ *m* the space group of diamond. Each Eu^II^ cation in EuADC is surrounded by eight oxygen atoms of the carboxylate groups of six different ADC^2−^ ligands. Two carboxylate groups coordinate in a bidentate chelating mode and four monodentately. The Eu^II^O_8_ polyhedron is best described as a snub diphenoid (J_84_ = JSD‐8, CShM=6.725).^20^ However, the large CShM value (continuous shape measures)^20^ already indicates that this polyhedron is highly distorted. Each Eu^II^O_8_ polyhedron is connected by common edges to four further Eu^II^O_8_ polyhedra resulting in a 3D framework structure with open channels. These channels are filled with the carbon atoms of the ADC^2−^ linkers. On the other hand, each linker coordinates to six Eu^II^ cations, three by each carboxylate group in a chelating, bridging (μ_3_‐η^1^:η^2^:η^1^) mode. The overall coordination can be described by the Niggli formula 3∞ [Eu(ADC)_6/6_]. A ToposPro analysis^21^ including the Eu^2+^ nodes and the connecting ADC^2−^ linkers leads to the rare unimodal *msw* topology accounting for 6‐connected vertices and two different kinds of edges.^22^

### Magnetic Properties

**Figure 4:**
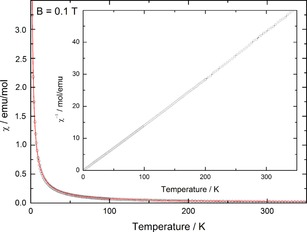
Magnetic susceptibilities and inverse magnetic susceptibilities (inset) of EuADC measured at *B*=0.1 T as a function of temperature. The red line shows the least squares fit to the data (see text).

To confirm the divalent oxidation state of europium in EuADC temperature‐dependent magnetic susceptibilities were measured in an external field of 0.1 T. The resulting *χ*=f(*T*) and *χ* ^−1^=f(*T*) curves are shown in Figure 4.

The linear curve in the *χ* ^−1^=f(*T*) plot indicates a Curie–Weiss behaviour for an (almost) pure paramagnet. From a Curie–Weiss fit according to Equation (3):

(3) $$\chi \left(T\right)=C/\left(T-\theta {}_{P}\right)+\chi {}_{\mathrm{dia}}$$

with *χ*_dia_=−67×10^−6^ emu mol^−1^ representing the diamagnetic contribution of EuADC, *θ*_P_=−0.31 K and an effective magnetic moment *μ*_eff_=7.76 μ_B_ were obtained. The very small (negative) *θ*_P_ value points to an almost pure paramagnetism with negligible antiferromagnetic interactions. These interactions are obviously much weaker than in for example, EuC_2_, where *θ*_P_=17.1 K and ferromagnetic interactions were observed.^23^ This indicates that ADC^2−^ is only a weak ligand for magnetic exchange interactions, as the Eu‐Eu distances are comparable in both compounds: 4.435 Å (4×) in EuADC vs. 4.124–4.410 Å (10×) in EuC_2_.^[23a)]^ The effective magnetic moment *μ*_eff_=7.76 μ_B_ in EuADC is close to the expected value *μ*_eff_=7.94 μ_B_ for the half‐filled 4f‐shell of Eu^2+^ with *J*=*S=*7/2 and *g*=2 according to *μ*_*s*.o._=2$\sqrt{S(S+1)}$ μ_B_. For Eu^3+^ ([4f^6^], ground multiplet ^7^F_0_), a completely different magnetic behaviour with a van Vleck susceptibility is expected^24^ so that the occurrence of larger amounts of Eu^3+^ in EuADC can be ruled out.

### ^151^Eu Mössbauer Spectroscopy

**Figure 5:**
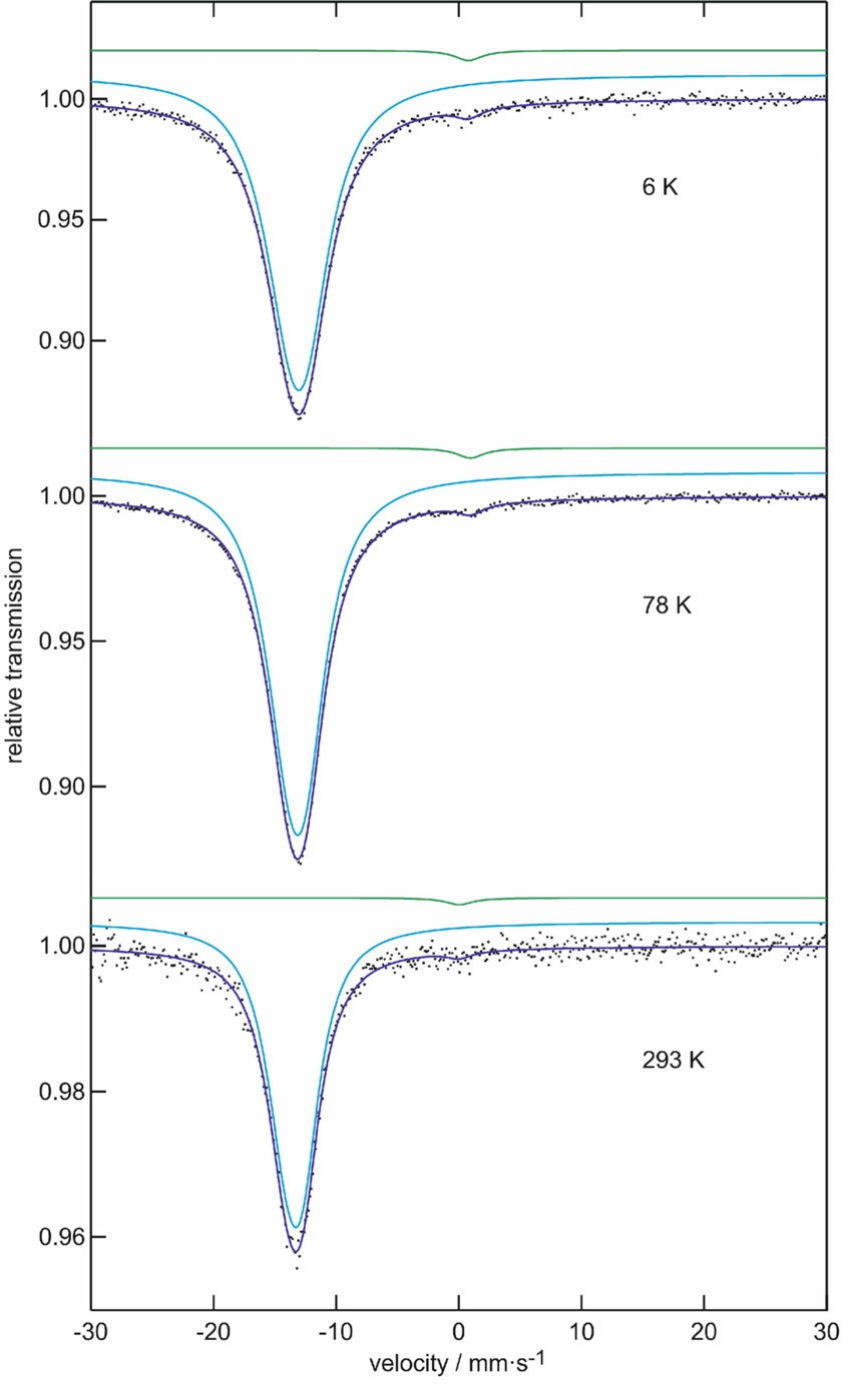
Experimental (data points) and simulated (continuous lines) ^151^Eu Mössbauer spectra of EuADC at different temperatures.

The ^151^Eu Mössbauer spectra of EuADC at 6, 78, and 293 K are presented in Figure 5; the corresponding fitting parameters are listed in Table 2. EuADC shows a main signal at an isomer shift of −13.25 mm s^−1^ (78 K data), indicating divalent europium and a highly ionic bonding situation. Similar negative isomer shifts are typically observed for divalent europium halides, halide glasses or borate glasses.^25^, ^26^, ^27^ The tiny second signals around *δ*=0 mm s^−1^ indicate an Eu^3+^ contribution, most likely arising from trace amounts of surface oxidation products. These signals (around 1–2 % of the total area) were included as a simple Lorentzian within the fitting procedure.

**Table 2:** Fitting parameters of ^151^Eu Mössbauer spectroscopic measurements of EuADC at 6, 78, and 293 K; *δ*=isomer shift, Δ*E_Q_*=electric quadrupole splitting, *Γ*=experimental line width. Parameters marked with an asterisk were kept fixed during the fitting procedure.

*T* [K]	*δ* [mm s^−1^]	Δ*E*_Q_ [mm s^−1^]	*Γ* [mm s^−1^]	Ratio
6	−13.14(1)	3.4(2)	4.89(6)	98.4
	0.8(2)	0*	2.7*	1.6
78	−13.25(1)	3.46(7)	4.29(3)	98.5
	0.9(1)	0*	2.7*	1.5
293	−13.44(2)	3.4(2)	3.52(9)	98.6
	0.0(5)	0*	2.7*	1.4

The signals of divalent Eu^II^ were all symmetrical and we obtained stable fits without any constraints resulting in the line width and electric quadrupole splitting parameters listed in Table 2. The numerical values as well as a superposition of the spectra (not shown here) clearly reveal a weak broadening of the signal from ambient temperature down to 6 K. It is well known that for such weak broadening effects one observes correlation between the line width and the quadrupole splitting parameter, hampering a doubtless separate refinement. As a test we tried a fit of the 6 K spectrum with the line width fixed at the room temperature value. However, the simulated signal did not reproduce the experimental spectrum well. Thus, the broadening of the signal most likely relies on a distribution of slightly different quadrupole splittings.

To test the local coordination of each Eu^II^ cation in dependence of the temperature we refined the synchrotron powder diffraction data obtained at the ESRF and DELTA down to 100 K using the Rietveld method. The resulting structural parameters were analysed using the SHAPE algorithm.^20^ However, no clear trend of the CShM values for a JSD‐8 polyhedron was obtained in dependence of the temperature. A possible reason for the increasing line widths with decreasing temperatures in the Mössbauer spectra might be related to the negative thermal expansion observed in EuADC (see below). This effect is connected to a strongly increasing vibrational motion of the connecting oxygen atom (“guitar string vibration”)^28^ with increasing temperature. Such a motion will “smear” out the coordination around a central metal cation thus leading to a virtually higher symmetric coordination sphere. However, to confirm this assumption a more detailed structural analysis is necessary, which should be based on X‐ray single crystal data. Up to now, we have been unable to obtain single crystals suitable for such an analysis.

### Thermal Analysis

**Figure 6:**
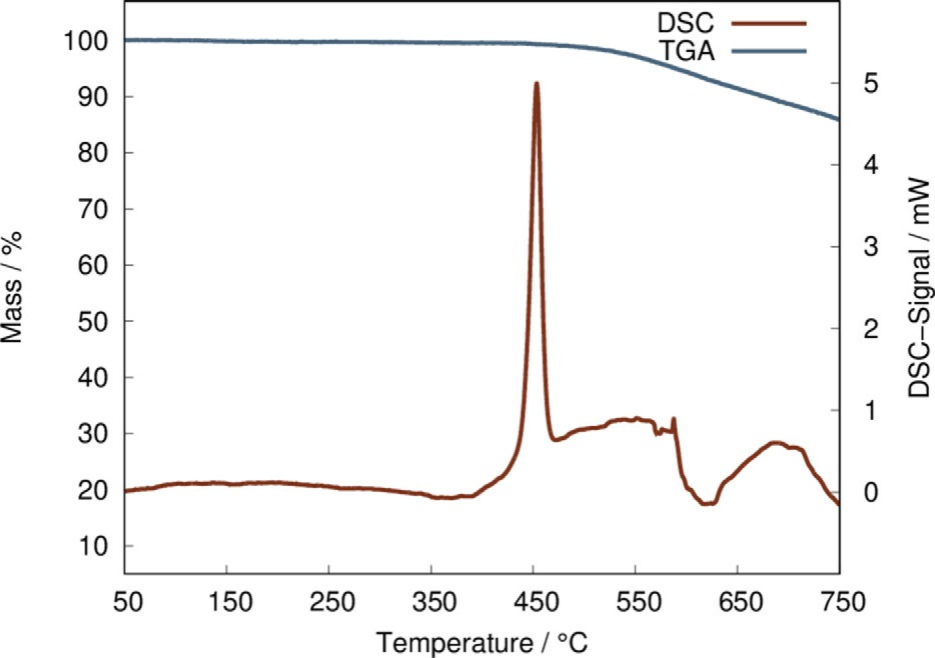
DSC (red) and TGA curve (blue) of EuADC (section up to 750 °C).

As already mentioned, EuADC shows a surprisingly high chemical stability for an Eu^II^ containing compound, as even after exposure to (humid) air for several months no signs of degradation were observed in the XRPD patterns. To examine the thermal stability of EuADC, DSC/TGA measurements were performed in an argon atmosphere up to 1000 °C. In Figure 6 the range up to 750 °C is depicted. The complete measurement is given in Figure S13, Supporting Information. EuADC shows a sharp exothermic signal at approx. 440 °C pointing to a decomposition of the material. This decomposition temperature is only slightly lower than that reported for SrADC.^12^ In Figure S14 (Supporting Information) the DSC/TGA curves of EuADC and SrADC are compared, measured under the same conditions. Accordingly, the decomposition temperature in SrADC is about 15 °C higher than in EuADC. A decomposition temperature of ca. 440 °C for an Eu^II^ containing coordination polymer is still remarkable, but not completely unprecedented.^29^, ^30^ However, for recently discovered ADC‐based MOFs^4^ much lower decomposition temperatures were reported.

For SrADC the decomposition of the material upon heating was thoroughly investigated: SrADC (=SrC_4_O_4_) releases CO upon heating and forms amorphous carbon as well as SrCO_3_. The latter decomposes to SrO at higher temperatures releasing CO_2_.^12^ For EuADC the release of CO should lead to a mass loss of 10.6 %, which was reached at approx. 630 °C, but with no clear plateau. Even up to 1000 °C no clear plateau was observed (Figure S13, Supporting Information). To clarify this point, several samples of EuADC were heated to 500, 700, and 1000 °C, and the resulting residues were investigated by XRPD. For the two lower temperatures (500 and 700 °C) only an amorphous solid was obtained, whereas for the residue obtained after heating to 1000 °C the XRPD pattern clearly shows the reflections of both modifications of Eu_2_O_3_ (Figure S15, Supporting Information). Obviously, SrADC and EuADC show a different decomposition reaction.

### Thermal Expansion

**Figure 7:**
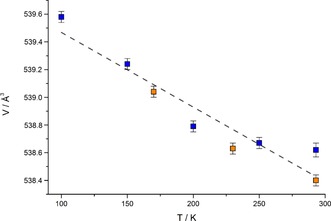
Unit cell volume of EuADC as a function of temperature. Blue squares indicate the values obtained upon cooling from room temperature to 100 K and orange squares those obtained upon heating from 100 K back to room temperature.

As mentioned in the introduction, SrADC shows a negative thermal (volume) expansion below room temperature. However, the resulting thermal expansion coefficient is small (*α*_V_=−4.7(13)×10^−6^ K^−1^)^12^ compared to other NTE materials.^31^ In this respect, it was interesting to see how the substitution of Sr^2+^ by Eu^2+^ in isotypic compounds with very similar unit cell dimensions affects the NTE properties. In Figure 7 the unit cell volumes of EuADC as obtained from temperature‐dependent synchrotron powder diffraction data (beamline BL9, DELTA, Dortmund/Germany) are plotted. All lattice parameters are listed in Table S2 (Supporting Information) and the respective Le Bail fits are given in Figures S3–S10. It should be noted that at the SNBL (ESRF, Grenoble/France) with a significantly better resolution a decomposition of EuADC after several hours within the high‐flux beam of this beamline was observed, as indicated by a black colour of the material exposed to the beam. Furthermore, the lattice parameters obtained at room temperature before and after cooling show large deviations, which make these measurements unsuitable for a precise determination of the temperature‐dependence of these lattice parameters. Within the lower flux of the DELTA synchrotron also a slight blackening of EuADC after several hours within the synchrotron beam was observed, but the lattice parameters are still reproducible, as can be seen in Figure 7.

The unit cell volumes plotted in Figure 7 clearly indicate a negative thermal volume expansion in EuADC. From a linear fit between 293 and 100 K, a negative thermal expansion coefficient *α*_V_=−9.4(12)×10^−6^ K^−1^ was obtained, which is significantly larger than in SrADC (*α*_V_=−4.7(13)×10^−6^ K^−1^).^12^ As in both compounds the occurrence of NTE is attributed to transverse M^II^⋅⋅⋅O⋅⋅⋅M^II^ vibrations (“guitar string vibrations”),^28^, ^32^ its thermal expansion coefficient *α*_V_ will mainly depend on the flexibility of the structure, that is, the strength of the M⋅⋅⋅O bond. If an ionic character of this bond is assumed, its strength will depend on the M⋅⋅⋅O distances, as was pointed out by Kepert and co‐workers in a model they developed for NTE in a number of Prussian Blue analogues, M^II^Pt^IV^(CN)_6_ with M^II^=Mn, Fe, Co, Ni, Cu, Zn, Cd.^17^ Here, the absolute values of *α*_V_ increase with increasing size of the M^II^ cation. Accordingly, the *α*_V_ values in SrADC and EuADC should be very similar, as the M⋅⋅⋅O distances are almost the same in both compounds (cp. Table 3). The different masses of Sr^II^ and Eu^II^ should only have a minor influence. In agreement with this simple model Kepert and co‐workers found very similar *α*_V_ values in a series of LnCo(CN)_6_ materials for Ln=Y^III^ and Ho^III^, which have very similar ionic radii, but different masses.^33^ In the view of these results the different *α*_V_ values for SrADC and EuADC are surprising, but it should be noted that the determination of precise lattice parameters in this structure type is difficult, since the pseudo‐cubic symmetry (see *c*/*a* ratio in Table 3) leads to an overlap of many reflections of the tetragonal unit cell (cp. Figure 2). However, measurements on a high‐resolution synchrotron diffractometer are not applicable here, as these compounds tend to decompose in high‐flux synchrotron beams (see above). At the moment we are working to set up a laboratory X‐ray diffractometer with high resolution to examine this interesting class of compounds in more detail.

**Table 3:** Lattice parameters of M^II^ADC compounds crystallizing in the SrADC type structure, all determined at ambient conditions.

	*a* [Å]	*c* [Å]	*V* [Å^3^]	*c*/*a*	M⋅⋅⋅O [Å]
CaADC^14^	6.8778(3)	10.2011(4)	482.55(5)	1.483	2.3156(9), 4×; 2.728(2), 4×
SrADC^12^	7.210(1)	10.338(2)	537.5(1)	1.434	2.507(2), 4×; 2.751(2), 4×
EuADC^[this work]^	7.21668(8)	10.3165(1)	537.29(2)	1.430	2.501(3), 4×; 2.758(5), 4×
PbADC^13^	7.284(1)	10.325(2)	547.8(2)	1.417	2.559(9), 4×; 2.74(1), 4×

### Optical Spectroscopy

**Figure 8:**
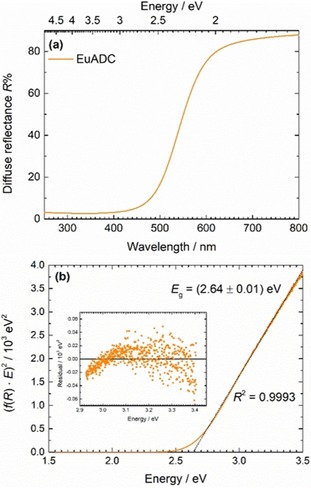
(a) (Diffuse) reflectance spectrum of EuADC and (b) Tauc plot of the spectrum indicating a direct band gap transition. The linear least‐squares fit is indicated as a dotted black line. Inset: Residual plot showing the deviations of the data from the linear fit in the fitting range.

Microcrystalline EuADC has an orange colour (Figure S2, Supporting Information). This appearance is in agreement with its diffuse reflectance spectrum that is shown in Figure 8 a and indicates an onset of increasing reflectance *R* above around 450 nm. The optical band gap was more accurately characterized with aid of a so‐called Tauc plot^34^, ^35^ (see Figure 8 b, upper panel). In there, f(*R*) denotes the Kubelka–Munk function^36^ that is related to the diffuse reflectance by Equation (4):

(4) $$f\left(R\right)=\frac{{\left(1-R\right)}^{2}}{2R}$$

and is a measure for the absorption coefficient of the powder assuming a constant scattering contribution. Consequently, the absorption cross section *σ*_abs_ is directly proportional to f(*R*)⋅*E*. It may then be easily concluded that above the optical band gap *E*_g_ for a direct band gap, a linear relation between (*f*(*R*) ^.^ *E*)^2^ and *E* should follow Equation (5):

(5) $$\sigma_{abs}\propto f\left(R\right)\cdot E\propto {\left(E-E_{g}\right)}^{\frac{1}{2}}$$

which is simply a consequence of the energy dependence of the electronic density of states (DOS) in a bulk solid and its proportionality to *σ*_abs_ by Fermi's golden rule. A linear least‐squares fit to the linear part of the Tauc plot and extrapolation to the abscissa allows the determination of a direct optical band gap with the value *E*_g_=2.64(1) eV (or *λ*_g_=470 nm). The statistical deviation of the data from the linear fit as indicated in the residual plot (see Figure 8 b) indicates the validity of the assumption of a direct band gap in contrast to an in first instance similarly accurately fitted indirect optical interband transition (see Figure S16, Supporting Information). The value of the band gap agrees perfectly with the observed complementary orange colour of solid EuADC. It should be noted that, for example, EuCl_2_ and EuBr_2_, are colourless compounds indicating much larger band gaps.

It is well‐known that Eu^II^ containing compounds are important phosphors with a large variety of different applications.^37^ The broad 4f^6^5d^1^→4f^7^ transition is dependent upon the ligand field around Eu^II^ and thus in the focus of many research groups world‐wide.^38^, ^39^, ^40^, ^41^, ^42^ In contrast to those typical findings, no Eu^II^‐related luminescence was observed in EuADC, when excited with different energies even at low temperatures (10 K). The absence of any emission due to the 4f^6^5d^1^→4f^7^ transition of Eu^II^ is probably caused by concentration quenching. A more detailed analysis (e.g. investigation of doped compounds) of this unexpected finding is underway.

## Conclusions

In summary, we have synthesized orange coloured Eu^II^–acetylenedicarboxylate (Eu[O_2_C‐C≡C‐CO_2_] = EuADC) as a single‐phase microcrystalline powder. It crystallizes in the SrADC type structure (*I*4_1_/*amd*, *Z=*4)^12^ and is stable under ambient conditions for at least several months. Upon heating in an inert atmosphere decomposition only starts at 440 °C, which is remarkable for an Eu^II^ containing compound. Measurements of the magnetic susceptibilities (*μ*_eff_=7.76 μ_B_) as well as ^151^Eu Mössbauer spectroscopy data confirm that exclusively Eu^2+^ cations exist in EuADC. Below room temperature EuADC shows a negative thermal (volume) expansion (NTE) with *α*_V_=−9.4(12)×10^−6^ K^−1^. This value is slightly larger than that found in SrADC (*α*_V_=−4.7(13)×10^−6^ K^−1^).^12^ This finding is in conflict with established models of NTE,^17^ as in both compounds similar M^II^−O bonds are found. However, a precise determination of lattice parameters is difficult for this class of compounds, as due to its pseudo‐cubic structures a strong overlap of reflections occurs. Therefore, further experiments with a high‐resolution set‐up using laboratory X‐ray sources are under way, as acetylenedicarboxylates tend to decompose in high‐flux synchrotron beams after long exposure times.

According to diffuse reflectance spectra, EuADC exhibits a direct optical band gap of *E*_g_=(2.64±0.01) eV thus explaining the orange colour of the solid. Surprisingly, no photoluminescence under UV irradiation even down to 10 K was observed. In order to investigate the influence of concentration quenching (Eu‐Eu distances: 4.43531(4) Å) and in order to make the Eu^II^‐based electronic states more localized in real space, we have started to synthesize solid solutions Eu_*x*_Sr_1−*x*_ADC. Results on this solid solution will be published in due course.

## Experimental Section

**Synthesis of EuADC**: EuBr_2_ (Sigma Aldrich, 99.9 %) and H_2_ADC (Sigma Aldrich, 95 %) were used as purchased. K_2_ADC was obtained by grinding 0.0981 g K(CH_3_COO) (1 mmol) with 0.0571 g H_2_ADC (0.5 mmol) in an agate mortar, until no smell of acetic acid was noticed anymore. The product was finally heated for several hours at 150 °C in an Ar stream. Its purity was checked by XRPD.^43^ Two syntheses were developed according to procedures (1) and (2).

Procedure 1): In a glovebox, 0.312 g EuBr_2_ (1 mmol) and 0.190 g K_2_ADC (1 mmol) were transferred to a Schlenk tube and mixed. At a Schlenk line, approx. 7 mL degassed H_2_O were added. After complete dissolution of the solids the initially colourless solution turned slowly to yellow and a yellow‐orange precipitate started to form. The reaction mixture was stirred for approx. 24 h and the resulting precipitate was filtered off under inert conditions (Argon atmosphere). The product was washed with degassed water several times to remove the KBr by‐product as completely as possible. But even after repeated washing cycles a minor amount of KBr (approx. 3.5 weight %) remained in the product, which was finally dried in a dynamic vacuum.

Procedure 2): In a glovebox, 0.312 g EuBr_2_ (1 mmol) was placed in a Schlenk tube and at a Schlenk line, approx. 5 mL degassed H_2_O were added. In a second Schlenk tube, 0.114 g H_2_ADC (1 mmol) were weighed in. After evacuating and flushing the Schlenk tube with argon three times, approx. 3 mL degassed water were added. After complete dissolution of both solids the H_2_ADC solution was transferred to the EuBr_2_ solution via a syringe. The reaction mixture was stirred for approx. 24 h and the resulting orange precipitate was filtered off under inert conditions (argon atmosphere) and dried in a dynamic vacuum. XRPD investigations revealed that a single‐phase product had formed. C_4_EuO_4_ (264.00): Calcd: C, 18.20 %; Found: C, 18.40 %, H, 0 %, N, 0 %. Additionally, IR and Raman spectra (Figures S11 and S12, Supporting Information) showed the expected signals for carboxylate groups and C−C triple bonds and compared very well with the spectrum obtained for SrADC thus confirming the successful synthesis of EuADC.

**Powder X‐ray diffraction**: To check the purity of the samples XRPD data were recorded with a Huber G670 diffractometer (Ge(220) monochromator, image plate detector, room temperature, MoKα_1_ radiation), ca. 60 min per scan. Samples were sealed in glass capillaries (*Ø*=0.3 mm) under inert conditions (argon filled glovebox) prior to all measurements.

**Synchrotron powder diffraction**: High‐resolution synchrotron powder diffraction data was recorded at the Swiss Norwegian BeamLine (SNBL, BM01B)^44^ at the European Synchrotron (ESRF, Grenoble/France). The wavelength was calibrated with a Si standard NIST 640c to 0.504477 Å. The diffractometer is equipped with six counting channels, delivering six complete patterns collected with a small 1.1° offset in 2*θ*. A Si(111) analyser crystal is mounted in front of each NaI scintillator/photomultiplier detector. Data was collected at 295 K with steps of 0.002° (2*θ*) and 100 ms integration time per data point leading to a recording time of 22 min per scan (4°≤2*θ*≤30°). For the final pattern 5 scans were added. Data from all detectors and scans was averaged and added to one pattern with local software.

As after long exposure times in the high flux beam of the ESRF a decomposition of EuADC was observed, indicated by a darkening of the material, temperature dependent diffraction data was collected at beamline BL9 of the DELTA synchrotron radiation facility, Dortmund,^45^ which has a significantly lower photon flux. The measurement was performed at selected temperatures between 293 and 100 K (N_2_ cryostreamer) with a wavelength of *λ*=0.826566 Å using a PILATUS100K detector (7°≤2*θ*≤30°, approx. 60 min for each scan). To ensure a reproducibility of the obtained lattice parameters and exclude possible effects of decomposition in the synchrotron beam, patterns were recorded upon cooling (293, 250, 200, 150, and 100 K) and heating (170, 230, and 293 K).

For all experiments EuADC was filled in glass capillaries (*Ø*=0.7 mm) and sealed under an argon atmosphere. The capillaries were mounted on spinning goniometers.

**Analysis and refinement of powder diffraction data**: The WinXPow software package^46^ was used for raw data handling and visual inspection of the data. A Rietveld refinement was conducted with GSAS^47^ using the high‐quality diffraction pattern of EuADC obtained at the ESRF at 295 K. The known crystal structure of SrADC^12^ was used as a starting model for the refinement. Subsequently six background parameters (shifted Chebyshev function), scale, zero shift, lattice parameters (*a*, *c*) and seven profile parameters (pseudo‐Voigt function including four parameters to account for the anisotropic peak broadening) were refined. In the final refinement cycles four positional and four isotropic temperature factors (*U*_iso_) were introduced leading to 25 variables in total. Including three soft constraints (C1−C1=1.20(5) Å, C1−C2=1.45(5) Å, C2−O1=1.25(5) Å) a smooth and slowly converging refinement was obtained. The resulting fit is shown in Figure 2 and selected details of the crystal structure, the measurement and the refinement are summarized in Table 1, fractional atomic coordinates and selected interatomic distances are given in Table S1 (Supporting Information). Diamond^48^ was used for the visualization of the crystal structure of EuADC (Figure 3) and Gnuplot 4.6^49^ for the plot of the refinement.

To determine the unit cell parameters of the data obtained at the DELTA synchrotron upon cooling (293 to 100 K) and heating back to room temperature precisely, Le Bail fits in Jana2006^50^ were performed using the following five variables in all refinements: tetragonal lattice parameters *a* and *c*, zero shift, and profile parameters *GW* and *LY* (pseudo‐Voigt). The background was subtracted manually before each refinement. The lattice parameters obtained from these Le Bails fits are summarized in Table S1 (Supporting Information) and the unit cell volumes are plotted in Figure 7. Plots of the Le Bail fits are shown in Figures S3‐S10 (all Supporting Information). They were visualized using Gnuplot 4.6.^49^

**Elemental analysis**: Elemental analysis of carbon, hydrogen, and nitrogen was conducted with a HEKAtech GmbH EuroEA 3000 Analyser.

**IR spectroscopy**: IR spectra were recorded with a PerkinElmer Spectrum 400 IR spectrometer (ATR module, diamond crystal). The resulting spectrum is given as Figure S11, Supporting Information.

**Raman spectroscopy**: Raman spectra of solid EuADC and SrADC (Figure S12, Supporting Information) were recorded at room temperature on a Bruker MultiRam Raman spectrometer (40 mW; Nd:YAG laser, germanium detector) in the spectral range 4000‐0 cm^−1^. The samples were filled in 1 mm capillaries and sealed under an argon atmosphere.

**Measurement of magnetic susceptibilities**: The magnetic susceptibilities of a pressed powder sample of EuADC (2.41 mg) were measured in a SQUID magnetometer (MPMS, Quantum Design) applying an external field of 100 mT. The diamagnetic contribution of EuADC was calculated according to the procedures described in the literature.^51^ As shown in Figure 4, the inverse susceptibilities follow a straight line suggesting pure paramagnetic behaviour.

**Mössbauer spectroscopy**: The 21.53 keV transition of ^151^Eu of a ^151^Sm:EuF_3_ source with an activity of 50 MBq (0.91 % of the total activity; *I*=7/2 to *I*=5/2 transition) was used for the Mössbauer spectroscopic experiments, which were conducted in transmission geometry. The measurements were carried out in a continuous flow cryostat system (Janis Research Co LLC) at 6, 78, and 293 K (Figure 5). The temperature was controlled by a resistance thermometer (±0.5 K accuracy). The EuADC sample was enclosed in a small PVC container at a thickness corresponding to about 20 mg Mössbauer active element cm^−2^. The spectra were fitted with the Normos‐90 software package.^52^

**DSC/TGA**: DSC/TGA measurements on EuADC were performed with a Mettler Toledo TGA/DSC 1 Star^e^ (Al_2_O_3_ crucible; Argon stream with 30 mL min^−1^; heating rate 10 °C min^−1^). A sample of 2.6961 mg was weighed out and handled under inert conditions (glovebox). The residue obtained after heating to 1000 °C was investigated by XRPD (Figure S15, Supporting Information).

**Reflectance spectrum**: Diffuse reflectance spectra were acquired with a Cary 5000 UV/vis spectrometer (Agilent) that detects reflectance between 200 and 2500 nm. The reflectance spectrum was corrected for the background with a Spectralon reference sample.

**Luminescence spectroscopy**: Room temperature and low temperature (10 K) photoluminescence excitation and emission spectra of EuADC were recorded with a Horiba Jobin Yvon FluoroMax‐3 FL3‐22 spectrometer equipped with a 450 W Xe lamp, double Czerny‐Turner monochromators in both the excitation and emission compartment and a photomultiplier tube sensitive to the visible range (R928P, Hamamatsu). Spectra at 10 K were acquired with a liquid He closed‐cycle cryostat (Janis Research Co LLC) attached to an external temperature control unit (Lake Shore). For luminescence measurements, the powdered sample was sealed in pre‐evacuated spectroscopically pure quartz ampoules.

If not noted otherwise, diagrams and plots are visualized with Origin 8.5.0.^53^

## Conflict of interest

The authors declare no conflict of interest.

## Supporting information

As a service to our authors and readers, this journal provides supporting information supplied by the authors. Such materials are peer reviewed and may be re‐organized for online delivery, but are not copy‐edited or typeset. Technical support issues arising from supporting information (other than missing files) should be addressed to the authors.

Supplementary
