# Supplementary material for: Eu(O2C‐C≡C‐CO2): An EuII Containing Anhydrous Coordination Polymer with High Stability and Negative Thermal Expansion
Source: Chemistry. 2020 Feb 11;26(12):2726–34. doi: 10.1002/chem.201904966 (PMC7065108; doi:10.1002/chem.201904966)
Supplement: Supplementary file 1 — Supplementary [file CHEM-26-2726-s001.pdf]

CHEMISTRY
A European Journal

Supporting Information

$Eu(O_2C\text{-}C{\equiv}C\text{-}CO_2)$: An Eu^{II} Containing Anhydrous Coordination Polymer with High Stability and Negative Thermal Expansion

Verena K. Gramm,[a] Daniel Smets,[a] Ireneus Grzesiak,[a] Theresa Block,[b] Rainer Pöttgen,[b] Markus Suta,[c, d] Claudia Wickleder,[c] Thomas Lorenz,[e] and Uwe Ruschewitz*[a]

chem_201904966_sm_miscellaneous_information.pdf

Supporting Information

$Eu(O_2C\text{-}C{\equiv}C\text{-}CO_2)$: An Eu(II) Containing Anhydrous Coordination Polymer with High Stability and Negative Thermal Expansion

Verena K. Gramm,[a] Daniel Smets,[a] Ireneus Grzesiak,[a] Theresa Block,[b] Rainer Pöttgen,[b] Markus Suta,[c,d] Claudia Wickleder,[c] Thomas Lorenz,[e] Uwe Ruschewitz*[a]

[a] Department of Chemistry, University of Cologne, Greinstraße 6, D-50939 Köln, Germany

[b] Institut für Anorganische und Analytische Chemie, WWU Münster, Corrensstraße 30, D-48149 Münster, Germany

[c] Institut für Anorganische Chemie, Universität Siegen, Adolf-Reichwein-Straße, D-57068 Siegen, Germany

[d] current affiliation: Dr. M. Suta, Debye Institute for Nanomaterials Science, Universiteit Utrecht, Pricetonplein 1, NL-3584 CC Utrecht, The Netherlands

[e] Institute of Physics II, University of Cologne, Zülpicher Straße 77, D-50937 Köln, Germany

Table S1: Fractional atomic coordinates, isotropic displacement parameters ($Å^2$) and selected interatomic distances of EuADC.

Table S2: Lattice parameters of the Le Bail fits of EuADC measured at BL 9 (DELTA synchrotron) at different temperatures.

Figure S1: XRPD pattern of the reaction product of H_2ADC with Europium electride in liquid ammonia (-78 °C).

Figure S2: Snapshot of EuADC as obtained from the reaction of H_2ADC with $EuBr_2$ in degassed water.

Figure S3: Le Bail fit of EuADC (BL 9, DELTA, 293 K, cooling).

Figure S4: Le Bail fit of EuADC (BL 9, DELTA, 250 K, cooling).

Figure S5: Le Bail fit of EuADC (BL 9, DELTA, 200 K, cooling).

Figure S6: Le Bail fit of EuADC (BL 9, DELTA, 150 K, cooling).

Figure S7: Le Bail fit of EuADC (BL 9, DELTA, 100 K, cooling).

Figure S8: Le Bail fit of EuADC (BL 9, DELTA, 170 K, heating).

Figure S9: Le Bail fit of EuADC (BL 9, DELTA, 230 K, heating).

Figure S10: Le Bail fit of EuADC (BL 9, DELTA, 293 K, heating).

Figure S11: IR spectrum of EuADC.

Figure S12: Raman spectra of SrADC and EuADC.

Figure S13: DSC and TGA curves of EuADC (up to 1000 °C).

Figure S14: DSC and TGA curves of SrADC and EuADC (up to 750 °C).

Figure S15: XRPD pattern of the residue obtained after heating EuADC in the DSC/TGA to 1000 °C.

Figure S16: Tauc plot of the reflectance spectrum of EuADC assuming an indirect band gap transition.

Table S1. a) Fractional atomic coordinates, isotropic displacement parameters (Å^2) and b) selected interatomic distances (Å) of EuADC as obtained from Rietveld refinements measured at the SNBL, Grenoble/France (295(2) K, λ = 0.504477 Å).

**a)**	***x***	***y***	***z***	U_{iso}
**Eu**	0	1/4	3/8	0.0109(3)
**C1**	0	1/4	-0.06682(8)	0.048(3)
**C2**	0	1/4	0.0738(2)	0.065(5)
**O1**	0.1536(4)	1/4	0.1302(4)	0.038(2)
**b)**				
**Eu – Eu**	4.43531(4), 4x		**C1 – C1**<br>**C1 – C2**	1.201(2)[A]<br>1.451(2)[A]
**Eu – O**	2.501(3), 4x<br>2.758(5), 4x		**C2 – O1**	1.252(2), 2x[A]

[A] soft constraints

Table S2. Lattice parameters of the Le Bail fits of EuADC measured at BL 9 (DELTA synchrotron, λ = 0.826566 Å) at different temperatures.

	***T* / K**	***a* / Å**	***c* / Å**	***V* / Å^3**
**cooling**	293	7.2233(4)	10.3231(6)	538.62(5)
	250	7.2239(3)	10.3223(4)	538.67(4)
	200	7.2249(3)	10.3218(5)	538.79(4)
	150	7.2275(3)	10.3230(5)	539.24(4)
	100	7.2296(3)	10.3236(5)	539.58(4)
**heating**	170	7.2264(3)	10.3222(5)	539.04(4)
	230	7.2238(3)	10.3219(4)	538.63(4)
	293	7.2224(3)	10.3215(4)	538.40(4)

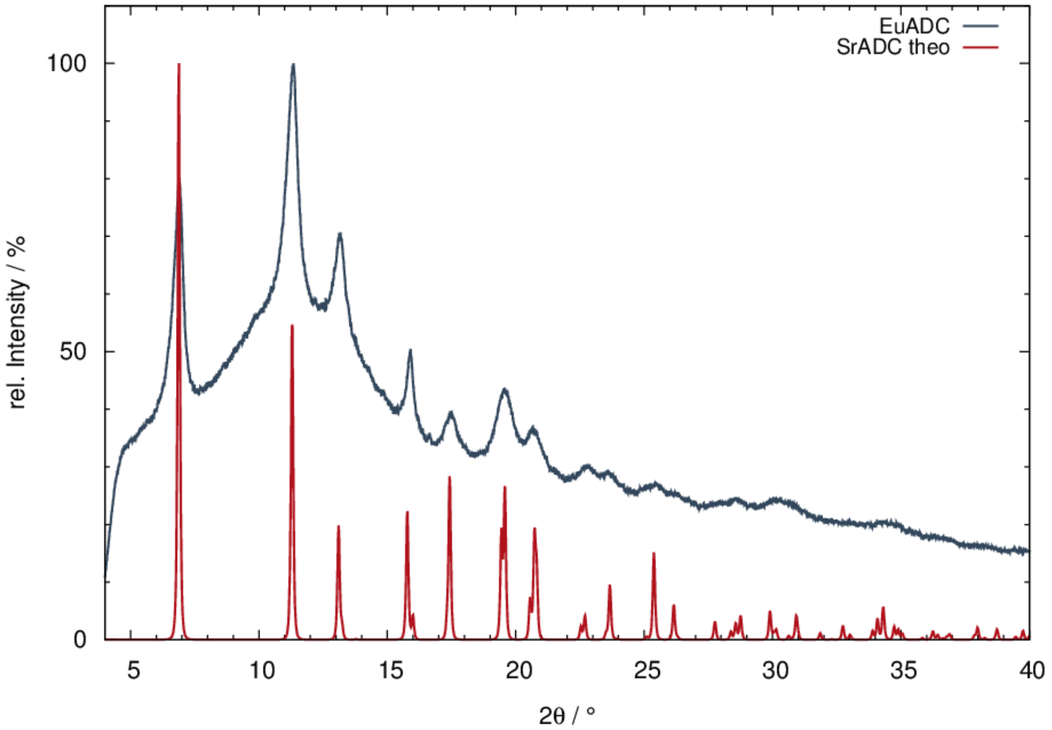

Figure S1. XRPD pattern (Huber G670, MoKα_1 radiation) of the reaction product of acetylenedicarboxylic acid (H_2ADC) with europium electride in liquid ammonia (approx. -78 °C). A pattern calculated from the known crystal structure of SrADC (red curve) is shown for comparison.

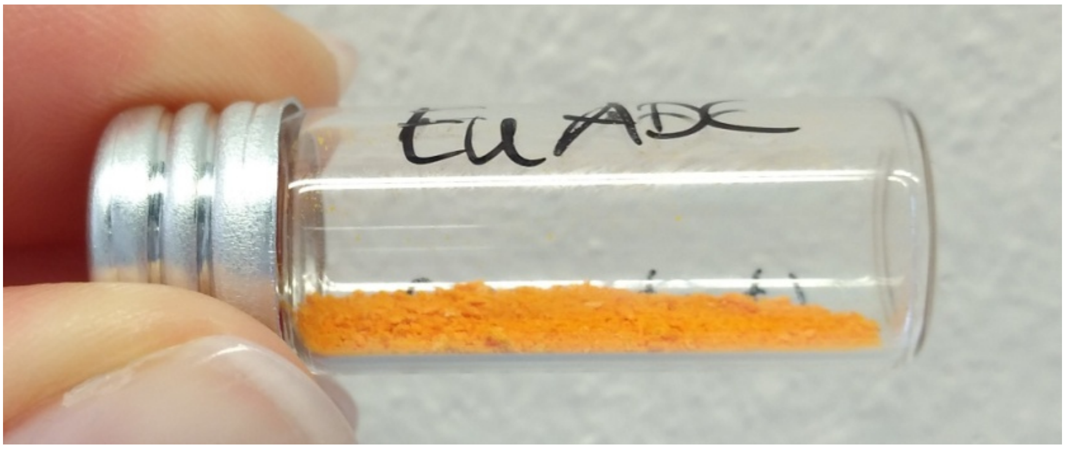

Figure S2. Snapshot of EuADC as obtained from the reaction of H_2ADC with $EuBr_2$ in degassed water.

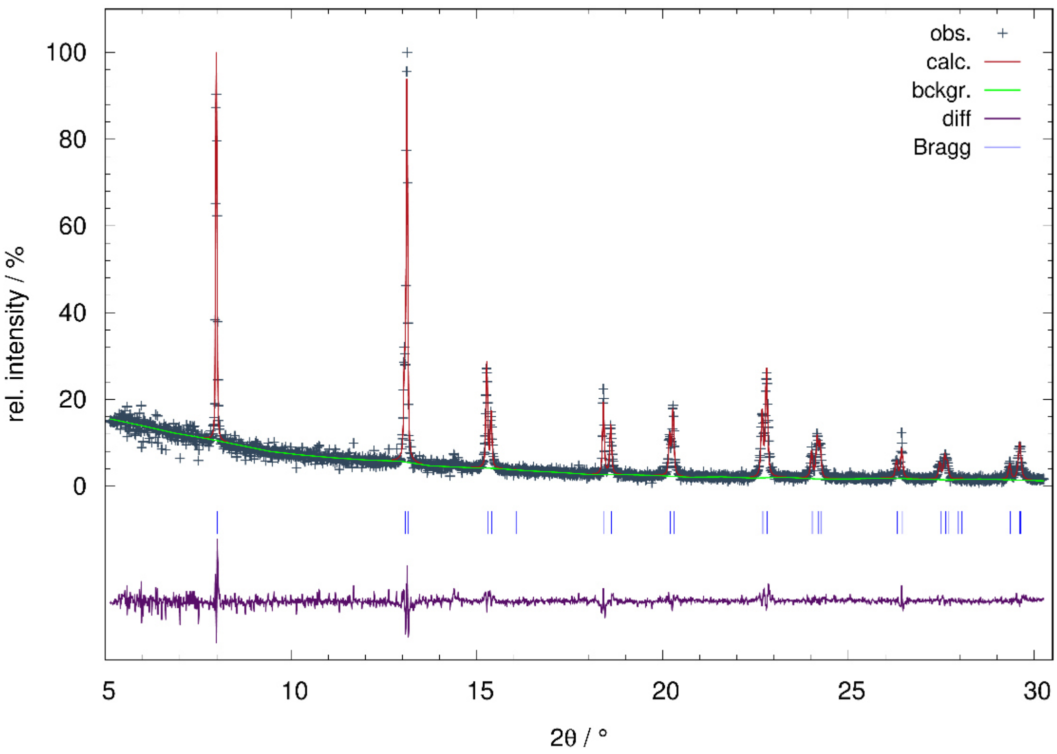

Figure S3. Le Bail fit of EuADC (BL 9, DELTA, 293 K, cooling).

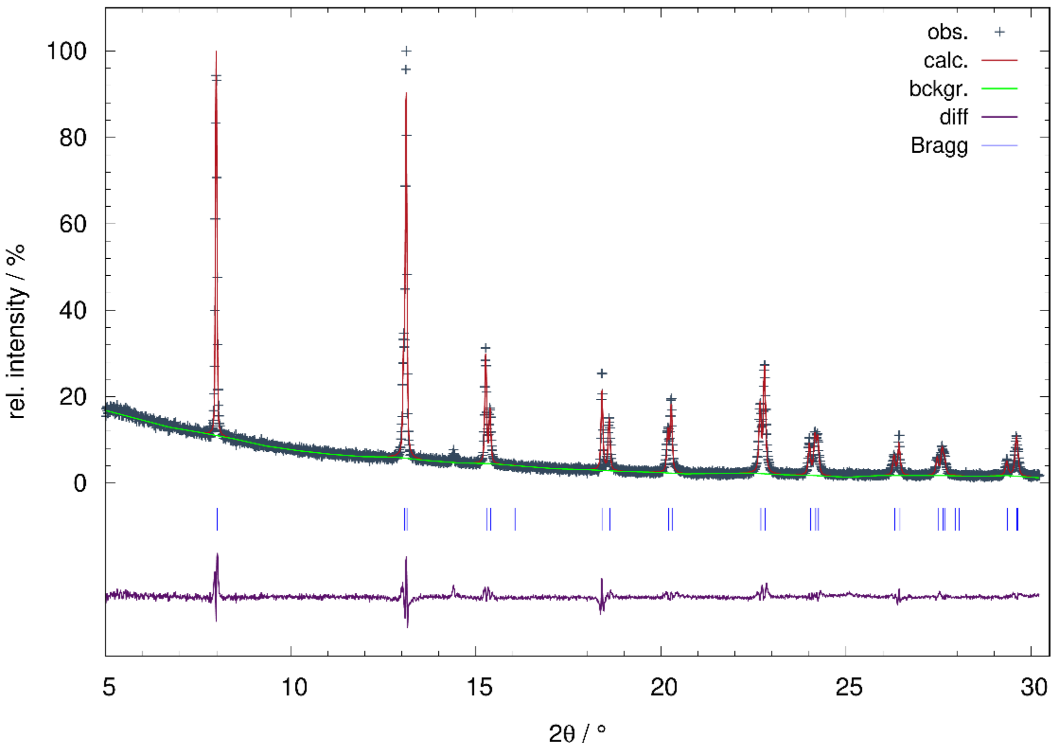

Figure S4. Le Bail fit of EuADC (BL 9, DELTA, 250 K, cooling).

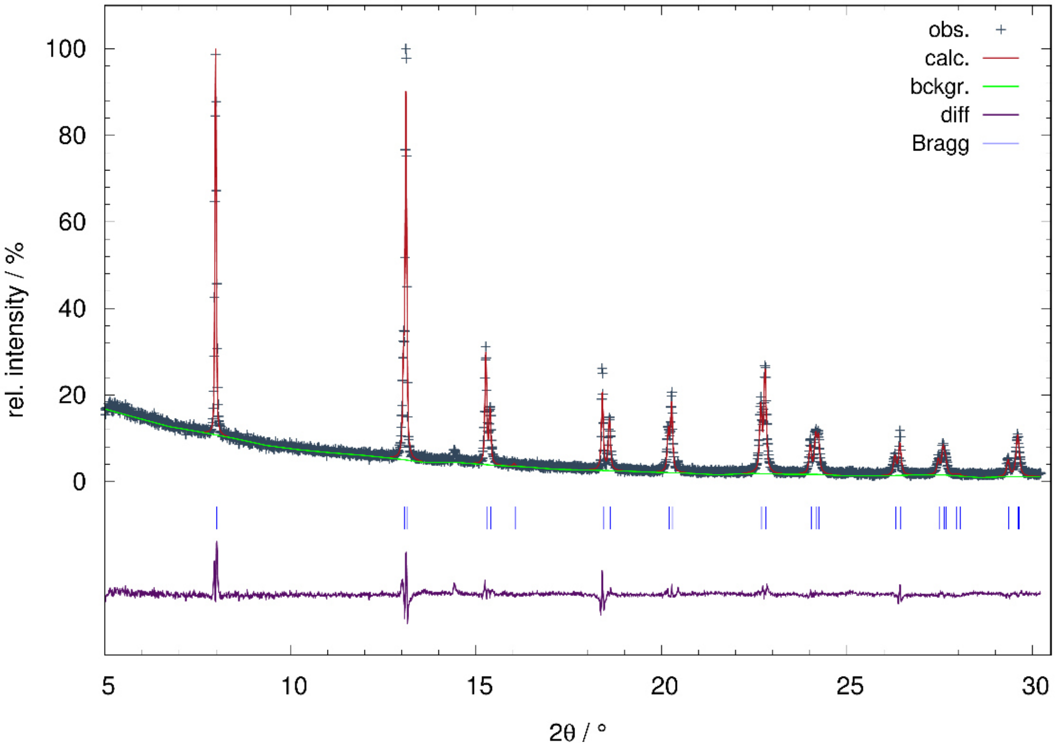

Figure S5. Le Bail fit of EuADC (BL 9, DELTA, 200 K, cooling).

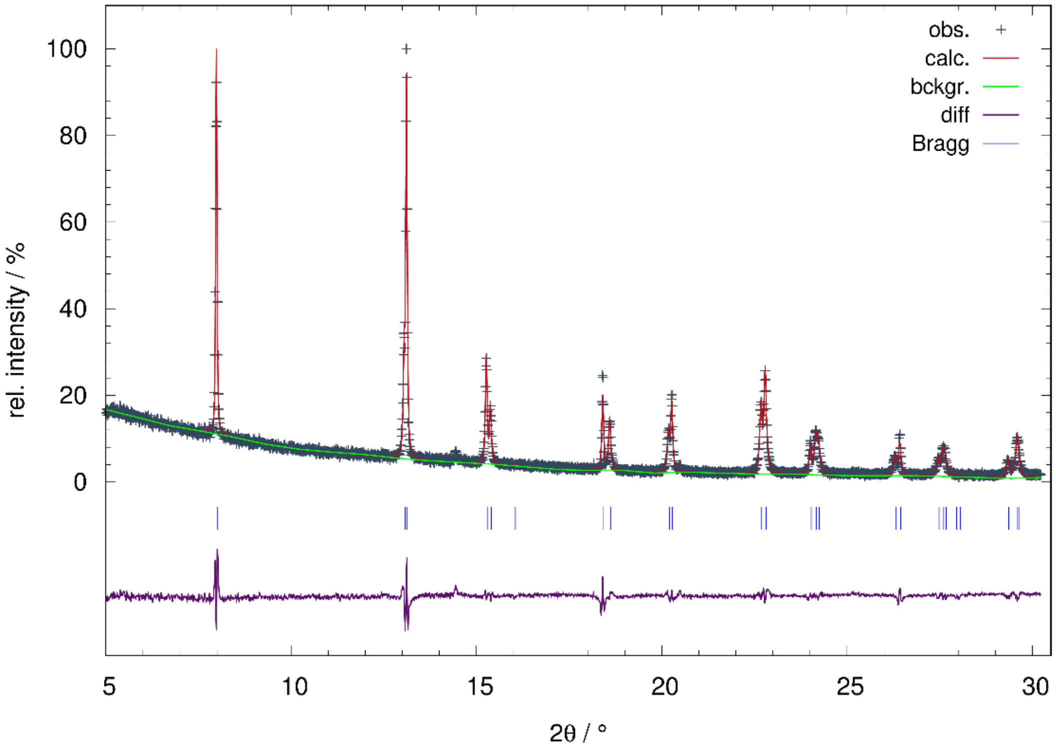

Figure S6. Le Bail fit of EuADC (BL 9, DELTA, 150 K, cooling).

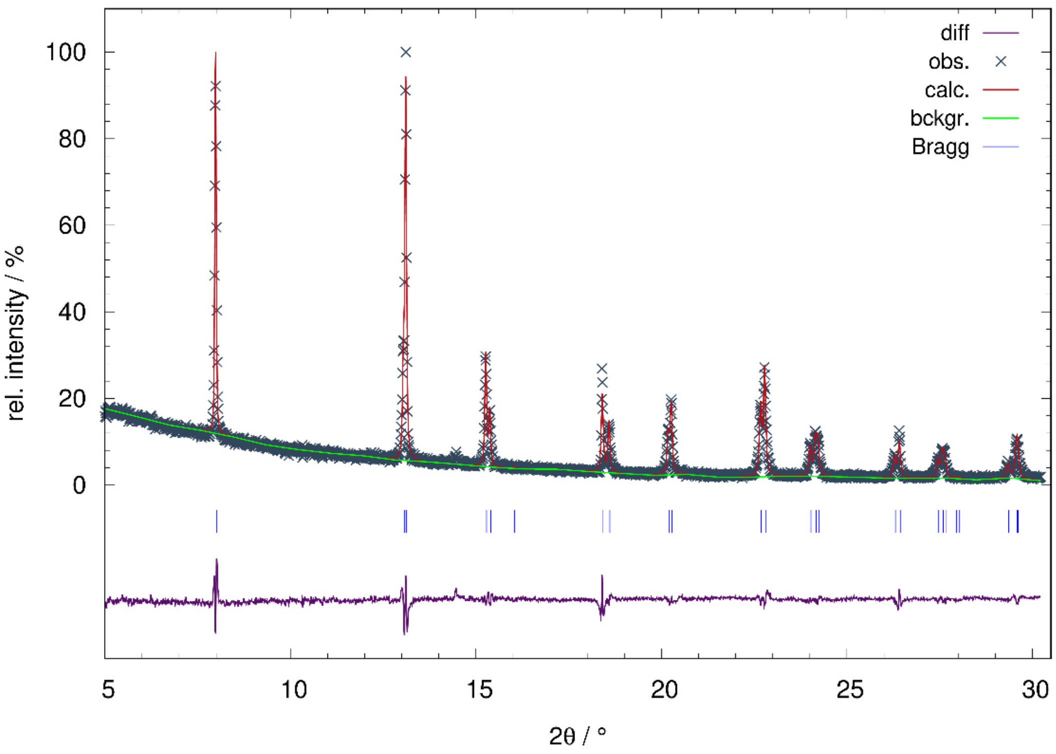

Figure S7. Le Bail fit of EuADC (BL 9, DELTA, 100 K, cooling).

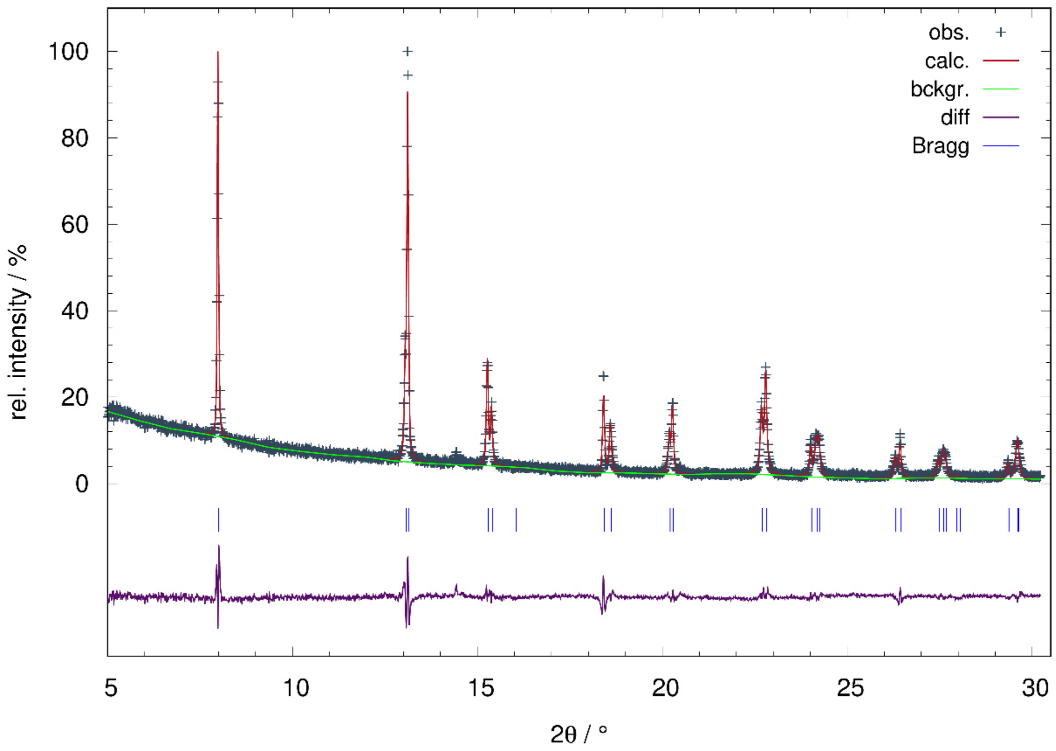

Figure S8. Le Bail fit of EuADC (BL 9, DELTA, 170 K, heating).

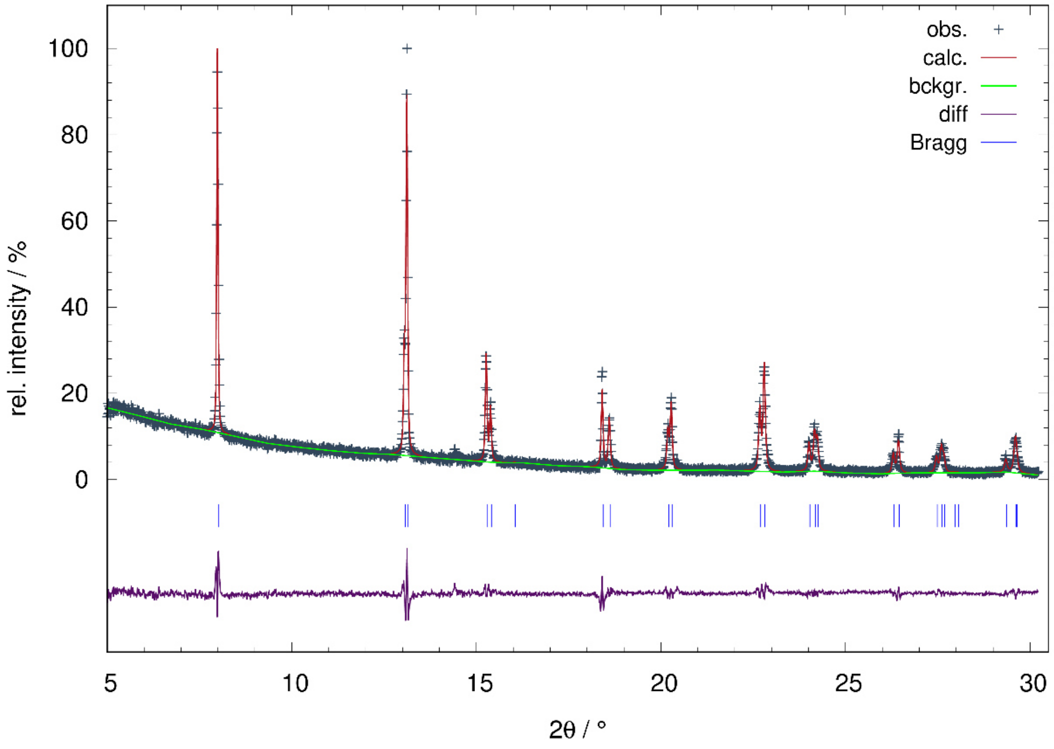

Figure S9. Le Bail fit of EuADC (BL 9, DELTA, 230 K, heating).

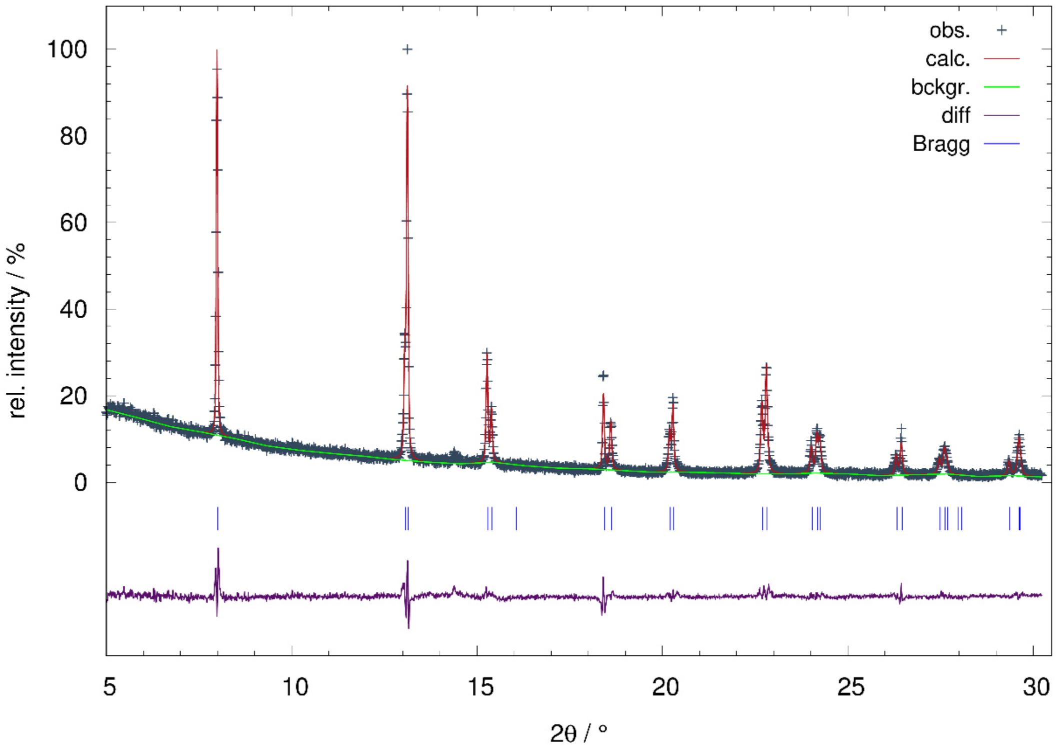

Figure S10. Le Bail fit of EuADC (BL 9, DELTA, 293 K, heating).

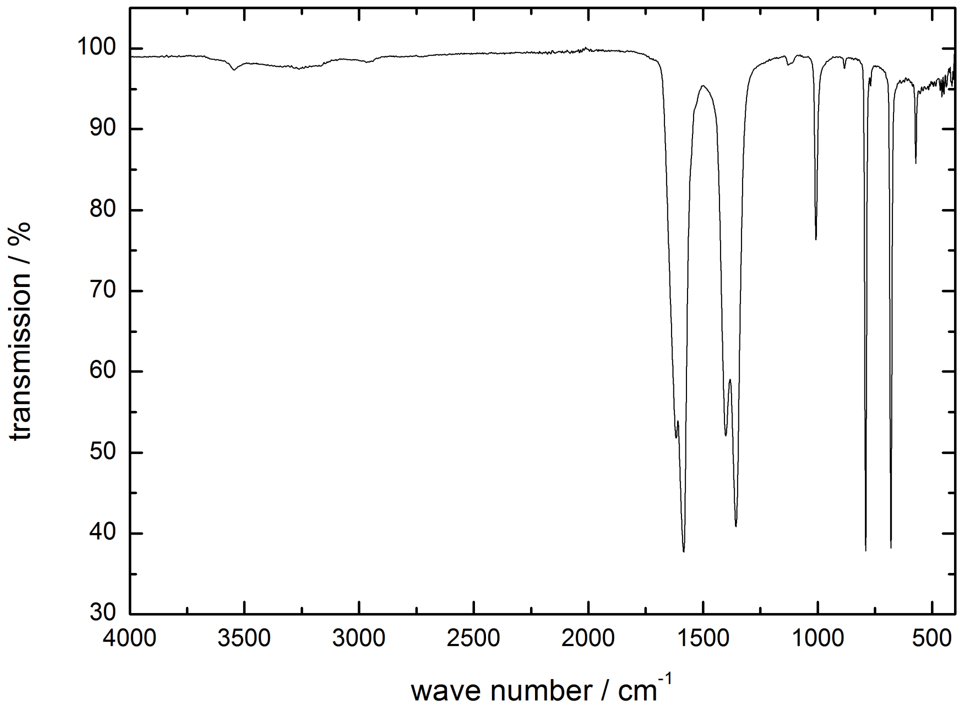

Figure S11. IR (ATR-MIR) spectrum of EuADC.

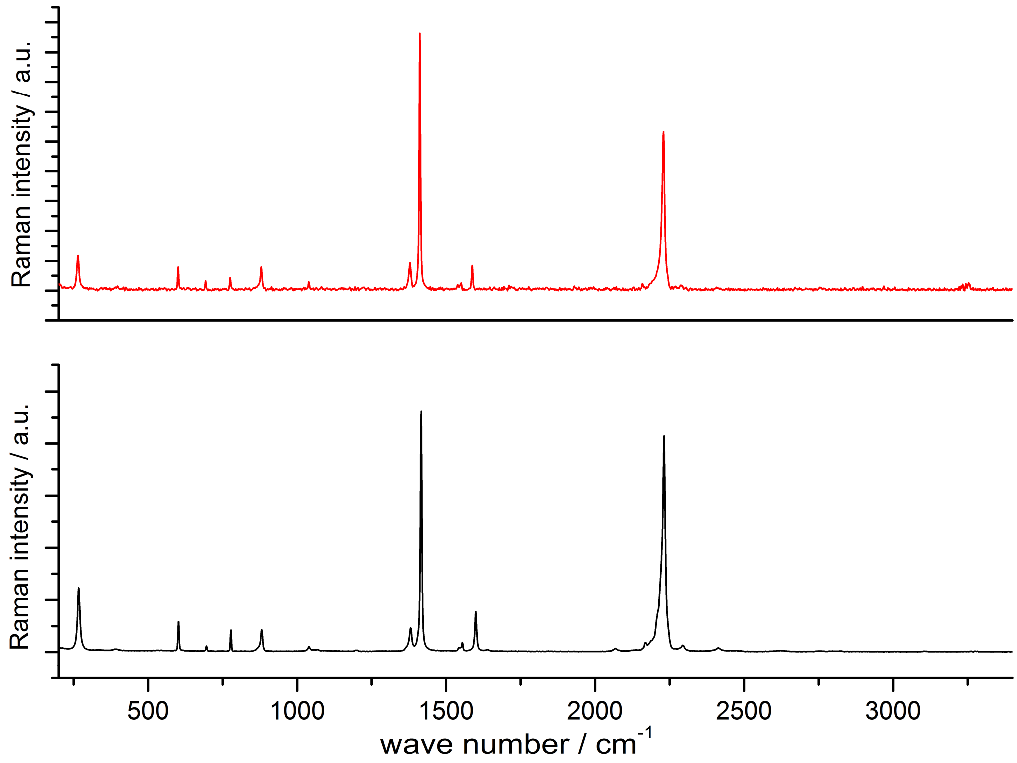

Figure S12. Raman spectra of SrADC (black) and EuADC (red).

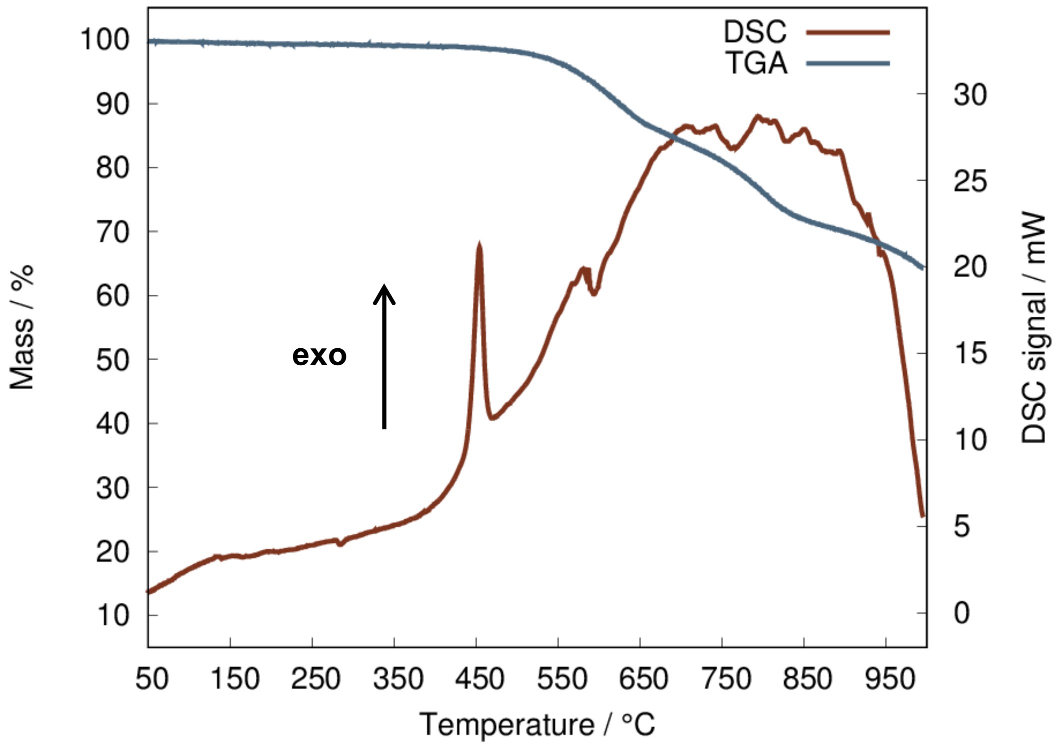

Figure S13. DSC (red) and TGA curves (blue) of EuADC (up to 1000 °C).

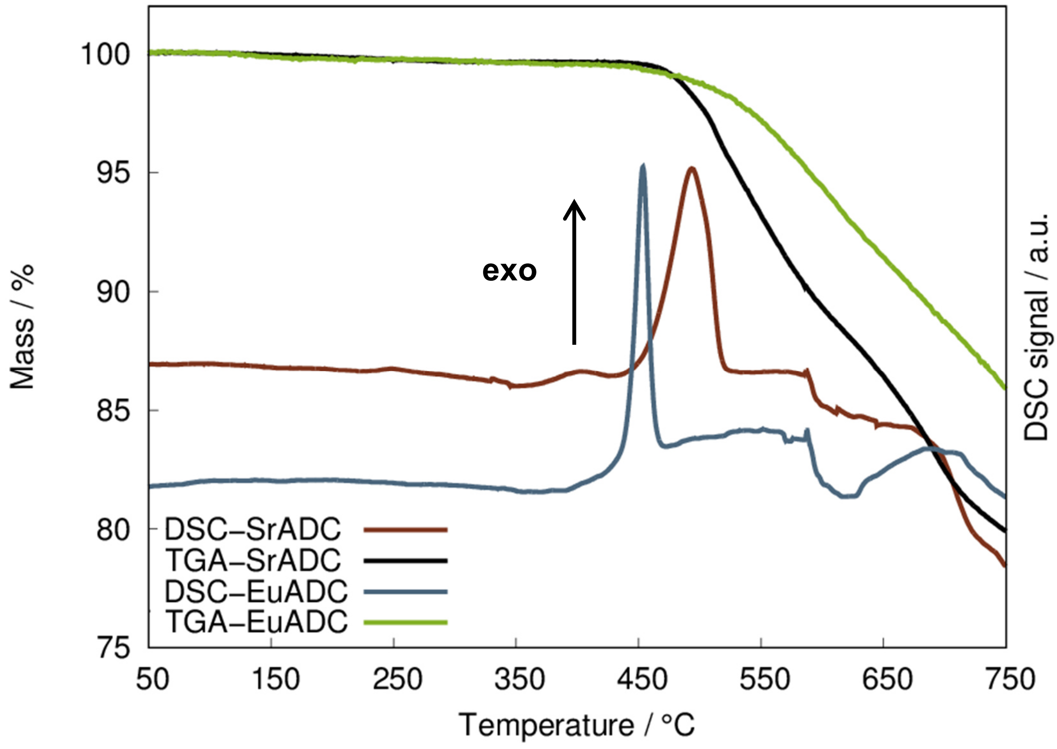

Figure S14. DSC and TGA curves of SrADC (red, black) and EuADC (blue, green) up to 750 °C.

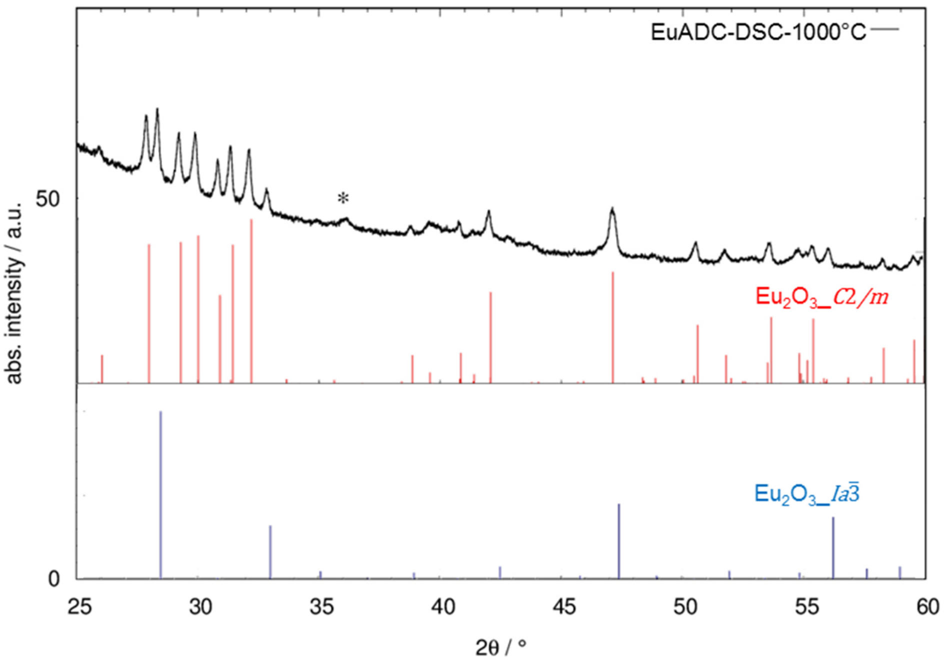

Figure S15. XRPD pattern of the residue obtained after heating EuADC in the DSC/TGA at 1000 °C (black curve). Line diagrams calculated from the known crystal structures of monoclinic Eu_2O_3 (*C*2/*m*, red line diagram) and cubic Eu_2O_3 ($Ia\bar{3}$, blue line diagram) are shown for comparison. A „*“ marks the reflection of the foil of the sample holder (Huber G670, Cu Kα_1 radiation, Ge(111) monochromator, image plate detector, 295(2) K, samples were measured as flat samples between two transparent foils).

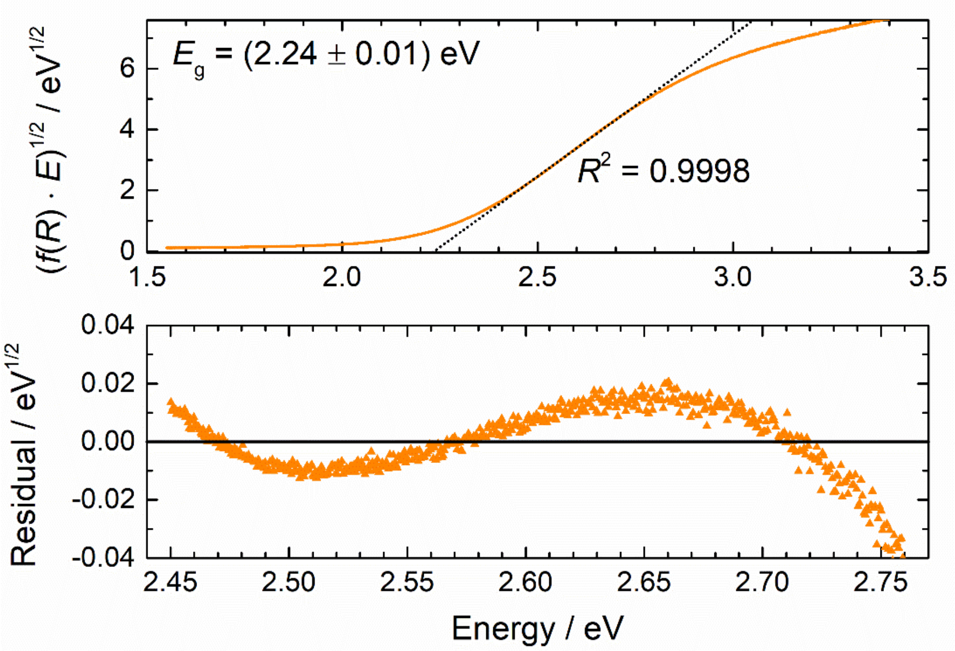

Figure S16. *Upper panel*: Tauc plot of the reflectance spectrum of EuADC assuming an indirect band gap transition. The linear least-squares fit is indicated as a dotted black line. *Lower panel*: Residual plot showing the non-statistical (!) deviations of the data from the linear fit in the fitting range.
